# Latent Profiles of Psychological Distress and Suicidal Ideation Among Nursing Students: A Cross‐Sectional Study of Associated Factors

**DOI:** 10.1155/da/9138758

**Published:** 2026-10-19

**Authors:** Anshi Wang, Jingjing Wan, Qin Yang, Lijun Zhu, Liying Wen, Weiwei Chang, Yu Zhu, Yan Chen, Yuelong Jin, Yingshui Yao

**Affiliations:** ^1^ School of Public Health Wannan Medical College Wuhu China wnmc.edu.cn; ^2^ School of Nursing Anhui College of Traditional Chinese Medicine Wuhu China

**Keywords:** latent profile analysis, nursing students, psychological distress, suicidal ideation

## Abstract

**Background:**

Psychological distress and suicidal ideation frequently co‐occur among nursing students, but their heterogeneity is often overlooked.

**Objectives:**

To identify latent profiles of nursing students based on psychological distress and suicidal ideation dimensions, and to explore factors associated with profile membership.

**Methods:**

A cross‐sectional survey of 1461 Chinese nursing students was conducted. Latent profile analysis (LPA) was applied to psychological distress and suicidal ideation dimensions to identify profiles, and multinomial logistic regression was used to examine factors associated with profile membership.

**Results:**

Three latent profiles emerged: C1: Low distress–low suicidal ideation (60.4%), C2: moderate distress–moderate suicidal ideation (30.8%), and C3: high distress–high suicidal ideation (8.8%). Multinomial regression revealed factors associated with C2/C3 vs. C1, including poor sleep quality, internet overuse/addiction, and moderate self‐efficacy. Factors associated with lower likelihood of C2 vs. C1 included good academic performance, moderate/high self‐esteem, and moderate/high time management skills. Factors associated with higher likelihood of C3 vs. C1 included higher body mass index (BMI), undergraduate institution enrollment, morning/neutral chronotypes, whereas exercise ≥1 time/week and a good parental relationship were associated with lower likelihood of C3 vs. C1.

**Conclusion:**

Nursing students exhibit three profiles, with 8.8% belonging to C3. Profile‐informed screening and preventive strategies considering factors associated with profile membership (sleep quality, internet addiction, exercise frequency, time management, self‐esteem, and self‐efficacy) and contextual stressors (institution type, BMI, and parental relationship) may inform future screening approaches for students exhibiting elevated psychological distress and suicidal ideation patterns.

## 1. Introduction

Depression, anxiety, and stress represent the most prevalent emotional problems and psychological disorders among university students [1]. These conditions frequently co‐occur [2] and exhibit high intercorrelations [3]. Concurrently, suicide remains among the leading causes of death among young people globally [4, 5]. Together, these mental health challenges constitute a growing public health crisis among university student populations worldwide. Recent evidence from the global burden of disease (GBD) 2023 analysis further demonstrates that mental disorders remain among the leading contributors to years lived with disability worldwide, with depressive and anxiety disorders continuing to account for a considerable proportion of the disease burden, particularly among adolescents and young adults [6]. These findings highlight the urgent need to strengthen early identification and prevention strategies targeting mental health problems among university populations.

Meta‐analytic evidence indicates the prevalence rates of anxiety and depression among university students at 26.0% and 27.0%, respectively [7]. Chinese students, in particular, experience substantial psychological stress, predominantly stemming from academic pressure within an examination‐oriented educational context [8]. Academic stress has been identified as an important contributor to mental health problems, including depression and anxiety, among Chinese adolescents [9]. Crucially, stress, anxiety, and depression have been associated with an elevated risk of self‐harm [10], suicide attempts [11], and suicidal ideation [5]. Studies suggest that between 8% and 18% of university students experience suicidal ideation, while lifetime suicide attempts range from 1% to 11% [12]. In the present study, suicidal ideation is conceptualized as thoughts, wishes, or cognitive representations related to ending one’s own life, whereas suicidal behavior refers to observable actions associated with suicide, including suicide attempts and completed suicide. Although suicidal ideation and suicidal behavior are closely related, they represent distinct constructs with different psychological mechanisms and clinical implications. Therefore, the present study focuses specifically on suicidal ideation rather than suicidal behavior, aiming to identify heterogeneous patterns of suicidal ideation in relation to psychological distress among nursing students.

Compared to general university students, nursing students appear to face an even greater risk of suicide [13, 14] and experience higher rates of mental health problems such as anxiety and depression [15, 16]. The unique nature of nursing education frequently exposes students to potentially traumatic and ethically challenging situations, including encounters with patient and family anxiety, death and dying, distressed patients and families, emergencies requiring rapid critical thinking, and learning within highly stressful clinical environments [17]. Furthermore, nursing students typically contend with demanding academic schedules, rigorous clinical and classroom expectations, and the profound stress inherent in confronting life, death, and suffering. These cumulative pressures can lead to significant emotional or psychological distress and heightened levels of mental strain [17]. A meta‐analysis revealed global prevalence rates of depression, anxiety, and stress among nursing students at 52%, 32%, and 30%, respectively [18]. Additionally, the core responsibility of nursing, which involves direct patient care, places substantial demands on emotional competencies. This heightened exposure can predispose nurses, and consequently nursing students, to adverse outcomes, including distress, depression, disability, and suicidal behavior, particularly among individuals lacking adequate coping skills or strategies [19]. However, suicidal ideation should not be interpreted as a direct consequence of psychological distress alone. Rather, it represents a complex phenomenon shaped by the interaction of individual vulnerability, subjective psychological suffering, interpersonal experiences, and broader sociocultural contexts. Given the substantial burden of psychological distress and suicidal ideation among nursing students, identifying the mechanisms underlying their co‐occurrence and heterogeneity is essential for developing effective prevention strategies. Although psychological distress and suicidal ideation frequently co‐occur, suicidal ideation involves additional cognitive‐affective processes beyond general emotional symptoms. Therefore, an integrated yet theoretically informed framework is warranted to understand how psychological symptoms emerge, interact, and vary across individuals.

To better understand such heterogeneity, recent advances in psychopathology research have increasingly challenged traditional categorical approaches that conceptualize mental health problems as discrete and independent disorders. Instead, contemporary dimensional and transdiagnostic frameworks emphasize that psychological symptoms exist along continua and reflect shared underlying processes involving affective, cognitive, and contextual factors, contributing to heterogeneous patterns of psychological functioning. The research domain criteria (RDoC) framework and the hierarchical taxonomy of psychopathology (HiTOP) framework provide complementary perspectives for understanding psychopathology by emphasizing dimensional symptom structures and cross‐cutting mechanisms that extend beyond traditional diagnostic boundaries [20–22]. These perspectives suggest that symptoms such as depression, anxiety, stress, and suicidal ideation may represent interconnected but distinguishable manifestations of broader psychological dysfunction rather than isolated conditions.

Furthermore, affective neuroscience perspectives highlight the importance of emotional processing and regulation in understanding mental health problems. Alterations in these processes may contribute to impaired cognitive‐affective functioning and increased vulnerability to psychological symptoms [23]. Consistent with Gross’s emotion regulation framework, difficulties in monitoring, evaluating, and modifying emotional responses represent a transdiagnostic mechanism linking stressful experiences with diverse mental health outcomes [24, 25]. Therefore, simultaneously examining psychological distress and suicidal ideation may provide a more comprehensive understanding of heterogeneous mental health patterns among nursing students. Building on these dimensional and psychological perspectives, stress‐vulnerability models further explain why individuals exposed to similar stressors may experience different psychological outcomes, providing a framework for understanding individual differences in suicide‐related vulnerability.

Among stress‐vulnerability perspectives, the stress–diathesis model represents one of the most influential theoretical frameworks for explaining how pre‐existing vulnerabilities interact with stressful life events (stressors) to contribute to psychological disorders and suicidal behavior [26]. This model provides a framework for understanding how environmental stressors may contribute to suicide‐related vulnerability through psychological distress. According to this model, contextual stressors (e.g., academic demands and clinical challenges) interact with individual vulnerabilities (e.g., poor coping skills) and may contribute to psychological distress and subsequent suicidal vulnerability [19]. A study suggests that genetic predisposition (diathesis) and real‐world environmental adversities (stressors) are associated with greater psychological distress, which may in turn be linked to suicidal outcomes [27]. Furthermore, a theoretical mapping of suicide risk during the COVID‐19 pandemic suggested that pre‐existing vulnerabilities (diathesis), such as mood disorders or substance use disorders, interact with pandemic‐specific stressors (e.g., confinement, fear of infection) and may be associated with elevated suicide risk [28]. While the stress–diathesis model explains how vulnerability and stress exposure may contribute to suicide‐related risk, contemporary ideation‐to‐action theories of suicide, including cognitive models of suicide, further explain why suicidal ideation may emerge through specific cognitive‐affective processes. The cognitive model of suicide posits that suicide risk arises from the interaction between negative emotional states and maladaptive cognitive patterns, such as hopelessness, perceived burdensomeness, and thwarted belongingness [29]. Crucially, research aligned with this model indicates that improvement in mood alone does not necessarily lead to a reduction in suicidal ideation, underscoring the need to assess suicide‐related cognitions independently from general distress [30]. This theoretical premise justifies the integrated yet distinct measurement of psychological distress and suicidal ideation in our study. Recent analyses have further highlighted the role of cognitive distortions in mediating the relationship between stressors and suicide risk, with key examples including catastrophic thinking, magnification, and mind‐reading [28]. For nursing students, the high‐pressure environment may foster such distortions, for instance, by magnifying academic failures or perceiving oneself as a burden on peers and instructors.

Despite this established nexus between distress and suicide risk, prevailing research methodologies exhibit critical limitations. Most studies examine psychological distress and suicide risk in isolation, neglecting their synergistic effects and potential shared mechanisms. Furthermore, traditional variable‐centered approaches (e.g., regression models) fail to capture the potential heterogeneity in how distress and suicidality manifest and co‐occur within populations. They overlook the existence of theorized profiles, such as those exhibiting “discrepant distress–suicide severity” or “synchronous high‐distress/high‐suicidal ideation profiles,” which have been observed in general populations. This methodological gap limits the ability to develop targeted screening and preventive strategies, which is especially relevant for nursing students, as early identification of profiles characterized by elevated psychological distress and suicidal ideation could facilitate prevention efforts.

Previous research has often examined psychological distress (e.g., using the depression anxiety stress scales‐21 [DASS‐21]) and suicidal ideation (e.g., using the positive and negative suicidal ideation inventory [PANSI]) separately when exploring potential associated factors. The present study addresses the aforementioned gaps by employing latent profile analysis (LPA) to integrate dimensions from both the DASS‐21 (assessing depression, anxiety, and stress) and the PANSI (assessing PANSI). LPA is a person‐centered approach capable of uncovering latent profile structures within complex data, a strength lacking in traditional variable‐centered methods [31]. This approach is consistent with dimensional models of psychopathology, which emphasize heterogeneity in symptom configurations rather than assuming uniform patterns of mental health problems. LPA can identify profiles (e.g., “high distress‐low suicidal ideation”), which may facilitate early screening and preventive efforts, whereas traditional methods may overlook statistically derived patterns of distress and suicidal ideation. This approach facilitates a nuanced exploration of the heterogeneity and interrelated characteristics of these constructs. The identification of specific profiles may inform the development of tailored, profile‐informed screening and preventive strategies within academic settings. Therefore, this study aimed to identify latent profiles of nursing students based on psychological distress and suicidal ideation dimensions and to examine factors associated with profile membership.

## 2. Materials and Methods

### 2.1. Study Design and Participants

This cross‐sectional survey was conducted from March to June 2024 among nursing students using a multistage sampling strategy involving convenience selection at the institution and class levels. First, two medical institutions were selected by convenience sampling: one vocational medical college (3‐year diploma program) and one undergraduate medical college (4‐year bachelor’s program). Second, all academic years were included per institution (years 1–3 in the vocational medical college; years 1–4 in the undergraduate medical college). Subsequently, convenience sampling was used to select classes from each academic year, with 6 classes per year sampled from the vocational college and 8 classes per year from the undergraduate medical college. All students in selected classes were invited to participate.

The minimum required sample size was calculated using the formula for cross‐sectional studies with categorical outcomes: *n* = *t* ^2^ *p*(1 − *p*)/*d* ^2^ (*t* = 1.96, *P* = prevalence of the outcome of interest, *d* = margin of error, defined as 10% of *P*). Based on preliminary survey data, the estimated prevalence rates were anxiety: 35%, depression: 30%, stress: 25%. To ensure adequate power for all measured outcomes, we adopted the most conservative estimate (*P* =  25%). Substituting these values, *n* = *t* ^2^ *p*(1 − *p*)/*d* ^2^ = 1.96^2^ × 0.25 × 0.75/(0.1 × 0.25)^2^ ≈ 400 × 0.75/0.25 = 1200. Accounting for a potential 20% nonresponse or invalid questionnaire rate, the adjusted minimum sample size was 1200/(1 − 0.20) = 1500. Ultimately, a total of 1550 participants were recruited to ensure robust statistical power.

This study recruited 1550 participants from 50 classes, achieving 100% questionnaire retrieval. Eligible participants were nursing students enrolled in the selected classes who voluntarily participated in the study. Before statistical analysis, invalid questionnaires were screened and excluded based on predefined criteria. Specifically, questionnaires with substantial missing responses (40 predominantly blank questionnaires) or systematic response patterns indicative of careless responding (49 questionnaires exhibiting patterns such as straight‐lining) were excluded. Finally, 1461 valid questionnaires were retained for analysis, resulting in a valid response rate of 94.26%. No item‐level missing data remained among the 1461 questionnaires included in the final analyses.

To ensure standardized questionnaire administration and minimize potential measurement error, a consistent data collection protocol was implemented across all participating classes. Before questionnaire completion, trained investigators provided a standardized verbal introduction explaining the study objectives, confidentiality principles, and requirements for completing the questionnaire. In addition, each scale included specific instructions describing the response format and completion requirements to facilitate accurate understanding of the items. All questionnaires were completed anonymously and independently under the supervision of trained investigators, and participants were encouraged to respond to each item based on their actual experiences.

### 2.2. Instruments

#### 2.2.1. Sociodemographic and Lifestyle Characteristics

General demographic information was collected using a questionnaire covering the following 17 items: age, gender (male/female), body mass index (BMI), grade, only‐child status (yes/no), hometown location (urban/town/rural), smoking status (yes/no), alcohol consumption (yes/no), weekly exercise frequency (≥30 min per session; categorized as 0 times, 1–2 times, 3–4 times, or ≥5 times), academic performance (poor: bottom 1/5 of grade ranking; moderate: middle 3/5; good: top 1/5), school club participation (yes/no), relationship with roommates (poor/moderate/good), parental relationship (poor/moderate/good), father’s educational level (low: junior high school or below; moderate: senior high school and technical secondary school; high: college or above), mother’s educational level (low: junior high school or below; moderate: senior high school and technical secondary school; high: college or above), family type (nuclear family [parents and children]; joint family [parents, children, and grandparents]; single‐parent family; reconstituted family), and institution type (vocational college/undergraduate college).

#### 2.2.2. DASS‐21

The DASS‐21 was developed by Lovibond et al. [32] and is widely used to assess symptoms of depression, anxiety, and stress. Previous psychometric studies have supported its construct validity by demonstrating its factorial structure representing depression, anxiety, and stress dimensions, while also providing evidence of concurrent validity through significant associations with established measures of depression, anxiety, and perceived stress [33]. The Chinese version of the DASS‐21 has also demonstrated satisfactory reliability and validity among Chinese populations, including university students [8, 34]. This 21‐item instrument comprises three dimensions (7 items per dimension): anxiety, depression, and stress. Each item is rated on a 4‐point Likert scale ranging from 0 (not at all) to 3 (almost always). Raw subscale scores are multiplied by 2 to obtain the final scores, with higher values indicating greater symptom severity in the respective domains. In this study, psychological distress was operationally defined by the DASS‐21 instrument, encompassing its three core dimensions: depression, anxiety, and stress symptoms. In the present sample, the DASS‐21 demonstrated strong internal consistency (Cronbach’s *α* = 0.951), with α coefficients of 0.881, 0.859, and 0.871 for the depression, anxiety, and stress subscales, respectively.

#### 2.2.3. PANSI

The PANSI was developed by Osman et al. [35] to assess positive and negative dimensions of suicidal ideation. Previous psychometric research has supported the factorial validity of the PANSI by confirming its two‐dimensional structure comprising PANSI. Evidence of convergent validity has also been demonstrated through theoretically expected associations with related psychological characteristics, including hopelessness and self‐esteem [36]. Furthermore, the PANSI has demonstrated satisfactory psychometric properties among university student populations, and its Chinese version has been applied in Chinese student samplesamong university student populations, and its Chinese version has been applied in Chinese student samplesamong university student populations, and its Chinese version has been applied in Chinese student samples [34, 36]. This 14‐item scale comprises two dimensions: positive suicidal ideation (PSI, reverse‐scored) and negative suicidal ideation (NSI). Positive suicidal ideation reflects protective cognitions related to life appreciation and future orientation rather than suicidal vulnerability. Therefore, scores on this dimension were reverse‐coded before inclusion in the LPA. After reverse coding, higher standardized scores indicated fewer protective cognitions and greater suicidal vulnerability, allowing this indicator to be interpreted in the same direction as NSI within the latent profiles. Each item is rated on a 5‐point Likert scale ranging from 1 (never) to 5 (always). The total suicidal ideation score is calculated by summing both dimension scores, with higher scores indicating greater severity of suicidal ideation. In the present sample, the PANSI demonstrated strong internal consistency (Cronbach’s *α* = 0.896), with α coefficients of 0.852 and 0.944 for the PANSI dimensions, respectively.

#### 2.2.4. PSQI

The Pittsburgh sleep quality index (PSQI) was developed by Buysse et al. [37] and has demonstrated satisfactory psychometric properties, including reliability and validity, across diverse populations. Previous studies have supported its applicability among university students [38, 39]. This 18‐item instrument assesses seven components: subjective sleep quality, sleep latency, sleep duration, sleep efficiency, sleep disturbances, use of sleep medication, and daytime dysfunction. Each component is scored 0–3, with global scores ranging from 0 to 21; higher scores indicate poorer sleep quality. Participants with global scores >5 were classified as poor sleepers [40].

#### 2.2.5. MEQ‐5

The morningness–eveningness questionnaire (MEQ‐5) was derived from the 19‐item MEQ developed by Horne and Ostberg [41], and evaluates chronotype. This 5‐item scale has demonstrated acceptable reliability and validity for assessing chronotype and has been validated in university student populations [42, 43] and produces total scores ranging from 4 to 25. Participants are classified into three chronotypes based on their scores: evening type (scores 4–11), neutral type (scores 12–17), or morning type (scores 18–25).

#### 2.2.6. CIAS‐R

The revised Chinese internet addiction scale (CIAS‐R) was developed by Bai and Fan [44] through revisions incorporating *the Chinese Internet Addiction Scale*, *the Internet Addiction Diagnostic Questionnaire*, *the Pathological Internet Use Scale*, and *the Symptom Checklist 90* to assess the severity of internet addiction. The CIAS‐R has demonstrated satisfactory psychometric properties, including reliability and validity, and has been widely applied among university student populations [45, 46]. This 19‐item scale employs a 4‐point Likert scale ranging from 1 (extremely inconsistent) to 4 (very consistent). Total scores range from 19 to 76, with higher scores indicating greater severity of internet addiction. Participants are classified into three groups based on their scores: the normal internet use group (scores 19–45), the internet overuse group (scores 46–52), and the internet addiction group (scores 53–76) [47]. In the present sample, the CIAS‐R demonstrated strong internal consistency (Cronbach’s *α* = 0.950).

#### 2.2.7. ATMD

The adolescence time management disposition inventory (ATMD) was developed by Huang and Zhang [48] through revisions incorporating *the Time Management Questionnaire*, *the Time Management Behavior Scale*, and *the Time Structure Questionnaire* to assess time management skills. The ATMD has demonstrated satisfactory psychometric properties for assessing time management disposition and has been widely applied among Chinese university student populations [49, 50]. This 44‐item instrument comprises three dimensions: time value perception, time monitoring, and time efficacy. Items are rated on a 5‐point Likert scale ranging from 1 (strongly disagree) to 5 (strongly agree). Total scores are calculated by summing all item responses, with higher scores indicating better time management skills. Participants were classified into three groups based on interquartile scores [51]: low time management skills (scores 44–132); moderate time management skills (scores 133–160); and high time management skills (scores 161–220). In the present sample, the ATMD demonstrated strong internal consistency (Cronbach’s *α* = 0.966).

#### 2.2.8. RSES

The Rosenberg self‐esteem scale (RSES) was developed by Rosenberg [52], and has demonstrated robust reliability and validity across populations, including university students [53, 54]. This 10‐item instrument includes five reverse‐scored items. Responses for each item are recorded on a 4‐point Likert scale ranging from 1 (strongly disagree) to 4 (strongly agree). Total scores range from 10 to 40, with higher scores indicating greater levels of self‐esteem. Participants were classified into three groups based on the scores: low self‐esteem (scores 10–27); moderate self‐esteem (scores 28–32); and high self‐esteem (scores 33–40) [54]. In the present sample, the RSES demonstrated strong internal consistency (Cronbach’s *α* = 0.847).

#### 2.2.9. GSES

The general self‐efficacy scale (GSES) was developed by Schwarzer and Jerusalem [55], and has demonstrated satisfactory reliability and validity across different populations, including university students [53, 56]. This 10‐item instrument utilizes a 4‐point Likert scale ranging from 1 (not at all true) to 4 (exactly true). Total scores range from 10 to 40, with higher scores indicating stronger self‐efficacy. Participants were classified into three groups based on the interquartile scores [51]: low self‐efficacy (scores 10–26); moderate self‐efficacy (scores 27–29); and high self‐efficacy (scores 30–40). In the present sample, the GSES demonstrated strong internal consistency (Cronbach’s *α* = 0.839).

### 2.3. Data Analysis

Data were analyzed using SPSS 26.0 and Mplus 8.0. Categorical variables are presented as frequencies and percentages. Continuous variables that violated the assumption of normality, as assessed by the Kolmogorov–Smirnov test, are presented as medians with interquartile ranges (IQRs).

LPA was performed using Mplus 8.0 to identify latent profiles of participants based on the 3 dimensions of the DASS‐21 (depression, anxiety, stress) and the two dimensions of the PANSI (positive suicidal ideation and negative suicidal ideation). Following best practices in LPA, all continuous indicators were standardized to *z*‐scores prior to model estimation to mitigate scaling artifacts. The analysis commenced with a single‐profile model, and the number of profiles was incrementally increased. For each latent profile model, 200 random sets of starting values were generated, followed by 10 final stage optimizations to reduce the likelihood of local maxima and improve model convergence. The best log‐likelihood value was replicated across random starts, indicating stable model estimation. Model fit was evaluated using the following indices [57]: (1) Information criteria: Akaike information criterion (AIC), Bayesian information criterion (BIC), and sample‐size adjusted Bayesian information criterion (aBIC), where lower values indicate better fit. (2) Entropy: Values >0.80 signify classification accuracy exceeding 90%, with higher values indicating a superior fit. (3) Likelihood ratio tests: The Lo–Mendell–Rubin adjusted likelihood ratio test (LMR) and the bootstrap likelihood ratio test (BLRT). A significant *p*‐value (*p*  < 0.05) for these tests suggests that the *k*‐profile model provides a significantly better fit than the (*k*–1)‐profile model. (4) Profile sample size: Each latent profile should comprise at least 5% of the sample, though a minimum of 3% has also been deemed acceptable [58]. The optimal profile solution was selected based on multiple criteria, including statistical fit indices, classification quality, profile size, parsimony, and practical interpretability. When models with additional profiles demonstrated improved statistical fit but reduced parsimony or interpretability, the final profile solution was determined by considering the balance between statistical fit and practical interpretability.

The three‐profile solution was selected as optimal based on these criteria. Participants were probabilistically assigned to distinct latent profiles based on psychological distress and suicidal ideation (LP‐PDSI) using maximum‐likelihood posterior probabilities. The derived LP‐PDSI profile variable was used as the dependent variable in subsequent analyses. The identified profiles were considered statistically derived classifications reflecting patterns of psychological distress and suicidal ideation within the sample, rather than clinical diagnoses or categorical entities. Finally, chi‐square tests (for categorical variables), Kruskal–Wallis tests (for non‐normally distributed continuous variables), and one‐way analysis of variance (ANOVA, for normally distributed continuous variables) were employed to compare demographic characteristics and scale scores across the identified profiles. Variables showing statistically significant differences across profiles in univariate analyses were subsequently entered into the multinomial logistic regression model as candidate variables. This variable selection strategy was used to limit model complexity given the number of candidate variables. Because univariate significance screening may exclude theoretically relevant covariates, the resulting estimates were interpreted as multivariable‐adjusted associations rather than as evidence of independent risk or protective effects. Multicollinearity among the included variables was assessed before multinomial logistic regression analysis using tolerance values and variance inflation factors (VIFs). During data entry, range checks were applied to each variable to ensure that all entered values fell within predefined valid ranges, thereby minimizing potential input errors. No statistical outlier removal procedure was performed in this study. For psychological measures, scores at the higher or lower ends of the valid scale ranges were retained because they may represent meaningful variations in psychological distress and suicidal ideation rather than erroneous responses. Statistical significance was set at *p*  < 0.05.

### 2.4. Ethical Considerations

This study strictly followed the Declaration of Helsinki guidelines and was approved by the Ethics Committee of School of Public Health, Wannan Medical College (Approval No. LL‐WK2024ZQNZ16). The following measures were implemented to ensure ethical integrity and participant welfare:

1. Informed consent and voluntary participation: Before data collection, a brief briefing was provided to all potential participants, outlining the purpose of the study, potential risks (including the sensitivity of the questions), and benefits. They were explicitly informed that their participation was entirely voluntary, that they had the right to refuse to participate without any negative consequences, and that they could withdraw from the study at any time without penalty. Written informed consent was obtained from all participants.
2. Anonymity and confidentiality: No personal identifiers (such as names, student IDs, or contact information) were recorded on the questionnaires. The paper questionnaires were distributed in the classroom. Prior to distribution, seat arrangements were systematically adjusted to leave an empty seat between each student (i.e., no adjacent seating in front, behind, to the left, or to the right), specifically to prevent unauthorized viewing of students’ private information. In terms of content layout, the questionnaires consistently feature questions on the left‐hand side, response sections on the right‐hand side, and a perforated line in the middle; only the right‐hand response sections were collected upon retrieval. Prior to data collection, participants were explicitly informed that there were no “correct” or “incorrect” answers, and that their data would be used solely for scientific research purposes.
3. Relationship with recruiters and mitigation of coercion: The questionnaires were distributed by student counselors. However, to minimize any potential feelings of coercion or bias, the researchers explicitly instructed these individuals to emphasize the voluntary nature of the study and to assure students that their decision to participate (or not) would not have any impact on their academic performance, course evaluation, or other university‐related matters.
4. Support for participants and management of distress: Given the sensitive topic, a detailed debriefing statement was provided to all participants at the end of the questionnaire. This included contact information for free, professional psychological counseling services (e.g., the university’s mental health center hotline). Participants were encouraged to seek support if they experienced any discomfort or distress due to the study.
5. Follow‐up and support resources: Although the anonymous design prevented direct identification and follow‐up of individual participants with elevated suicidal ideation, this design and the corresponding risk‐management procedures were reviewed and approved by the Ethics Committee before data collection. Therefore, individual‐level follow‐up or intervention based on questionnaire responses was not feasible. Instead, a preventive support strategy was adopted: participants were provided with information about professional psychological services and encouraged to seek help if they experienced significant psychological distress or suicidal ideation. This approach provided all participants with access to available support resources after questionnaire completion.

## 3. Results

### 3.1. LPA of Psychological Distress and Suicidal Ideation

LPA was performed to identify distinct profiles based on the three DASS‐21 dimensions (depression, anxiety, stress) and the two PANSI dimensions (positive suicidal ideation and negative suicidal ideation). As presented in Table 1, the 4‐profile solution was rejected due to a nonsignificant LMR (*p* = 0.261). Subsequently, models with increasing profiles were compared. From the 1‐ to 3‐profile solutions, consistent decreases were observed in the AIC, BIC, and aBIC, alongside entropy values exceeding 0.90. Although the 2‐profile model exhibited marginally higher entropy (0.922 vs. 0.914), the statistically significant LMR (*p*  < 0.001) and BLRT (*p*  < 0.001) for the 3‐profile solution indicated substantially improved fit over the 2‐profile model. The 3‐profile solution demonstrated robust classification accuracy, with an entropy value of 0.914. The average posterior probabilities for low distress–low suicidal ideation (C1), moderate distress–moderate suicidal ideation (C2), and high distress–high suicidal ideation (C3) were 0.968, 0.943, and 0.976, respectively, indicating high certainty of profile assignment. The classification probabilities are presented in Table 2. Although the four‐profile solution demonstrated improved information criteria values compared with the three‐profile solution (AIC = 16171.249, BIC = 16319.282, and aBIC = 16230.335), it was not retained as the final model. The Lo–Mendell–Rubin adjusted likelihood ratio test for the four‐profile solution was nonsignificant (*p* = 0.261), suggesting that adding an additional profile did not significantly improve model fit. Moreover, the four‐profile solution resulted in a small additional profile containing only 3.6% of participants and showed slightly reduced classification quality compared with the 3‐profile solution (entropy = 0.902 vs. 0.914). Therefore, the three‐profile solution was retained as the final model because it achieved a better balance between statistical fit, classification quality, profile size, and interpretability.

**Table 1:** Model fit for latent profile analysis of nursing students’ psychological distress and suicidal ideation.

Model	Number of free parameters	Log (L)	AIC	BIC	aBIC	Entropy	LMR (*p*)	BLRT (*p*)	Class probabilities (%)
1	10	−10362.850	20745.699	20798.568	20766.801	—	—	—	100.0
2	16	−8946.319	17924.638	18009.228	17958.401	0.922	<0.001	<0.001	0.774/0.226
**3**	**22**	**−8296.909**	**16637.818**	**16754.130**	**16684.242**	**0.914**	**<0.001**	**<0.001**	**0.604/0.308/0.088**
4	28	−8057.625	16171.249	16319.282	16230.335	0.902	0.261	<0.001	0.517/0.290/0.036/0.157

*Note:* Bold values represent the optimal latent profile model (3‐class model) selected in this study.

Abbreviations: aBIC, sample‐size adjusted Bayesian information criterion; AIC, Akaike information criterion; BIC, Bayesian information criterion; BLRT, bootstrap likelihood ratio test; LMR, Lo–Mendell–Rubin adjusted likelihood ratio test.

**Table 2:** Classification probabilities for latent profiles of psychological distress and suicidal ideation among nursing students.

Potential profile	Probability of belonging to a potential profile		
	C1	C2	C3
C1	0.976	0.024	<0.001
C2	0.062	0.931	0.007
C3	<0.001	0.035	0.965

Abbreviations: C1, low distress–low suicidal ideation; C2, moderate distress–moderate suicidal ideation; C3, high distress–high suicidal ideation.

Each latent profile was characterized and named according to its standardized scores across all dimensions. Class 1 (C1) consisted of 883 (60.4%) nursing students with the lowest scores across all five indicators (depression, anxiety, stress, positive suicidal ideation [reverse‐scored], and NSI) and was named the “low distress–low suicidal ideation profile.” Class 2 (C2) consisted of 450 (30.8%) nursing students with moderate scores across five indicators and was named the “moderate distress–moderate suicidal ideation profile.” Class 3 (C3) consisted of 128 (8.8%) nursing students with the highest scores on all indicators and was named the “high distress–high suicidal ideation profile.” Visual representation of profile characteristics is provided in Figure 1.

**Figure 1:**
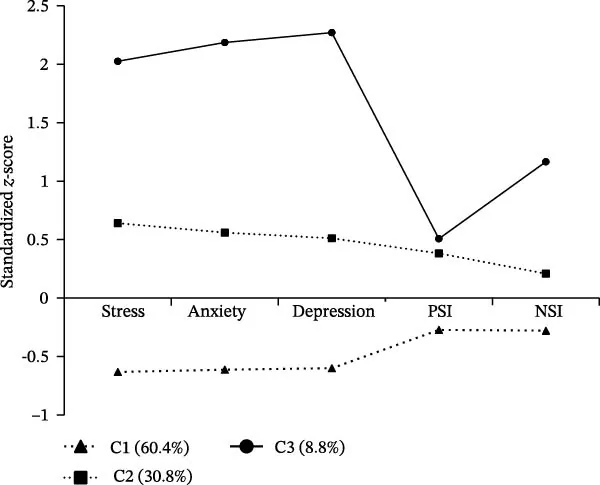
Three latent profiles of nursing students’ psychological distress and suicidal ideation based on standardized *z*‐scores. PSI, Positive suicide ideation (reverse‐scored); NSI, negative suicide ideation; C1, low distress–low suicidal ideation; C2, moderate distress–moderate suicidal ideation; C3, high distress–high suicidal ideation.

### 3.2. Characteristics and Univariate Analyses Across Latent Profiles

To examine heterogeneity across the latent profiles of psychological distress and suicidal ideation among nursing students, we conducted comparative analyses among the three latent profiles. Statistically significant differences were observed across all dimensions of psychological distress and suicidal ideation within the three‐profile solution (*p*  < 0.001, Table 3). Post hoc pairwise comparisons further indicated that while positive suicidal ideation showed significant differences only between C3 vs. C1 and C2 vs. C1, all other dimensions demonstrated statistically significant differences in every pairwise comparison (C3 vs. C2, C3 vs. C1, and C2 vs. C1). These differences consistently followed a graded pattern: C3 > C2 > C1 (all *p*  < 0.05). These findings demonstrate that the latent profile derived through LPA differentiated participants with varying levels of psychological distress and suicidal ideation, supporting the interpretability of the selected profile solution.

**Table 3:** Differences in profiles of nursing students in terms of five indicators (median [IQR]).

Variables	Total	Profiles			*H* ^a^	*p*‐Value
		C1	C2	C3		
Stress	6 (2, 14)	2 (0, 6)	14 (10, 14)	22 (20, 26)	994.262	<0.001
Anxiety	6 (2, 10)	2 (0, 4)	12 (8, 14)	22 (18, 24)	979.599	<0.001
Depression	4 (0, 10)	2 (0, 4)	10 (8, 14)	22 (18, 26)	983.751	<0.001
PSI	15 (12, 18)	13 (11, 17)	17 (14, 19)	18 (15, 20)	189.185	<0.001
NSI	10 (8, 17)	8 (8, 13)	13.5 (8, 18)	23 (16, 26)	267.523	<0.001

Abbreviations: C1, low distress–low suicidal ideation; C2, moderate distress–moderate suicidal ideation; C3, high distress–high suicidal ideation; NSI, negative suicide ideation; PSI, positive suicide ideation.

^a^Kruskal–Wallis test.

The final analytical sample comprised 1461 nursing students (median [IQR] age: 20 [18–20] years; BMI: 20.7 [19.0–22.9] kg/m^2^; 82.6% female; 20.5% only children; and 48.6% with rural hometowns). Undergraduate students constituted the majority (62.9%). The distribution across academic grades was relatively balanced, with Year 4 having fewer participants due to the absence of this grade in the vocational college program. A small proportion of participants reported alcohol consumption (12.5%) or smoking (3.5%). Over 80% engaged in exercise at least once per week. Most students (80.1%) reported moderate academic performance (middle 3/5 of grade ranking). Slightly over half (51.2%) participated in school clubs. Only 6.5% reported poor relationships with their roommates. Regarding family characteristics, the majority (72.1%) reported good parental relationships, and 68.8% came from nuclear families. Parental education levels were predominantly low, with 65.5% of fathers and 73.9% of mothers having attained junior high school or below. Results from specific scales revealed that more than half of the students (55.0%) were good sleepers (PSQI ≤ 5). Morning chronotypes (MEQ‐5) were uncommon (5.7%). The prevalence of internet addiction (CIAS‐R) was 10.0%, while 23.9% demonstrated high time management skills (ATMD). Over half of the students (58.9%) had low self‐esteem (RSES), and only 24.0% reported high self‐efficacy (GSES).

As shown in Table 4, the results of univariate analysis showed that nursing students across different LP‐PDSI profiles were distributed differently in terms of BMI, gender, only‐child status, smoking status, weekly exercise frequency, academic performance, relationship with roommates, parental relationship, institution type, sleep quality (PSQI), chronotype (MEQ‐5), internet addiction level (CIAS‐R), time management level (ATMD), self‐esteem level (RSES), and self‐efficacy level (GSES), and the differences were statistically significant (*p*  < 0.05 for all).

**Table 4:** Differences in profiles among nursing students in terms of general demographic information and the scales characteristic (*n* [%]).

Variables		Total	Profiles			*χ* ^2^/*H*	*p*‐Value
			C1	C2	C3		
Age (median [IQR])		20 (18, 20)	20 (19, 20)	20 (18, 20)	19.5 (18, 20)	1.123^a^	0.570
BMI (median [IQR])		20.7 (19.0, 22.9)	20.5 (19.1, 22.6)	20.5 (18.8, 22.9)	21.5 (19.6, 24.2)	13.174^a^	0.001
Grade	1	434 (29.7)	265 (30.1)	133 (29.6)	36 (28.1)	4.088	0.665
	2	425 (29.1)	258 (29.2)	122 (27.1)	45 (35.2)		
	3	390 (26.7)	229 (25.9)	130 (28.9)	31 (24.2)		
	4	212 (14.5)	131 (14.8)	65 (14.4)	16 (12.5)		
Gender	Male	254 (17.4)	131 (14.8)	83 (18.4)	40 (31.2)	21.479	<0.001
	Female	1207 (82.6)	752 (85.2)	367 (81.6)	88 (68.8)		
Only‐child status	Yes	300 (20.5)	166 (18.8)	97 (21.6)	37 (28.9)	7.414	0.025
	No	1161 (79.5)	717 (81.2)	353 (78.4)	91 (71.1)		
Hometown location	Urban	273 (18.7)	172 (19.4)	79 (17.5)	22 (17.2)	3.092	0.543
	Town	478 (32.7)	292 (33.1)	139 (30.9)	47 (36.7)		
	Rural	710 (48.6)	419 (47.5)	232 (51.6)	59 (46.1)		
Smoking status	Yes	51 (3.5)	21 (2.4)	21 (4.7)	9 (7.0)	9.854	0.007
	No	1410 (96.5)	862 (97.6)	429 (95.3)	119 (93.0)		
Alcohol consumption	Yes	183 (12.5)	106 (12.0)	56 (12.4)	21 (16.4)	1.981	0.371
	No	1278 (87.5)	777 (88.0)	394 (87.6)	107 (83.6)		
Weekly exercise frequency	0 times	250 (17.1)	128 (14.5)	90 (20.0)	32 (25.0)	14.252	0.027
	1–2 time	764 (52.3)	466 (52.8)	235 (52.2)	63 (49.2)		
	3–4 times	283 (19.4)	182 (20.6)	79 (17.6)	22 (17.2)		
	≥5 times	164 (11.2)	107 (12.1)	46 (10.2)	11 (8.6)		
Academic performance	Poor	128 (8.8)	49 (5.6)	61 (13.6)	18 (14.1)	36.814	<0.001
	Moderate	1170 (80.1)	719 (81.4)	357 (79.3)	94 (73.4)		
	Good	163 (11.1)	115 (13.0)	32 (7.1)	16 (12.5)		
School club participation	Yes	748 (51.2)	465 (52.7)	229 (50.9)	54 (42.2)	4.933	0.085
	No	713 (48.8)	418 (47.3)	221 (49.1)	74 (57.8)		
Relationship with roommates	Poor	95 (6.5)	51 (5.8)	31 (6.9)	13 (10.2)	52.128	<0.001
	Moderate	240 (16.4)	106 (12.0)	91 (20.2)	43 (33.6)		
	Good	1126 (77.1)	726 (82.2)	328 (72.9)	72 (56.2)		
Parental relationship	Poor	103 (7.0)	48 (5.4)	34 (7.6)	21 (16.4)	61.071	<0.001
	Moderate	305 (20.9)	141 (16.0)	124 (27.6)	40 (31.3)		
	Good	1053 (72.1)	694 (78.6)	292 (64.8)	67 (52.3)		
Father’s educational level	Low	957 (65.5)	571 (64.6)	306 (68.0)	80 (62.5)	2.188	0.701
	Moderate	303 (20.7)	186 (21.1)	87 (19.3)	30 (23.4)		
	High	201 (13.8)	126 (14.3)	57 (12.7)	18 (14.1)		
Mother’s educational level	Low	1078 (73.9)	648 (73.4)	344 (76.8)	86 (67.2)	5.437	0.245
	Moderate	233 (16.0)	145 (16.4)	64 (14.3)	24 (18.7)		
	High	148 (10.1)	90 (10.2)	40 (8.9)	18 (14.1)		
Family type	Nuclear family	1006 (68.8)	619 (70.1)	306 (68.0)	81 (63.3)	5.001	0.544
	Joint family	345 (23.6)	195 (22.1)	115 (25.6)	35 (27.3)		
	Single‐parent family	77 (5.3)	49 (5.5)	19 (4.2)	9 (7.1)		
	Reconstituted family	33 (2.3)	20 (2.3)	10 (2.2)	3 (2.3)		
Institution type	Vocational college	542 (37.1)	353 (40.0)	161 (35.8)	28 (21.9)	16.185	<0.001
	Undergraduate college	919 (62.9)	530 (60.0)	289 (64.2)	100 (78.1)		
PSQI	Poor sleeper	658 (45.0)	297 (33.6)	258 (57.3)	103 (80.5)	138.775	<0.001
	Good sleeper	803 (55.0)	586 (66.4)	192 (42.7)	25 (19.5)		
MEQ‐5	Evening type	400 (27.4)	241 (27.3)	132 (29.3)	27 (21.1)	10.180	0.038
	Neutral type	977 (66.9)	599 (67.8)	291 (64.7)	87 (68.0)		
	Morning type	84 (5.7)	43 (4.9)	27 (6.0)	14 (10.9)		
CIAS‐R	Normal	1034 (70.8)	744 (84.3)	254 (56.4)	36 (28.1)	237.507	<0.001
	Overuse	281 (19.2)	99 (11.2)	122 (27.2)	60 (46.9)		
	Addiction	146 (10.0)	40 (4.5)	74 (16.4)	32 (25.0)		
ATMD	Low	406 (27.8)	172 (19.5)	177 (39.3)	57 (44.5)	100.733	<0.001
	Moderate	706 (48.3)	441 (49.9)	206 (45.8)	59 (46.1)		
	High	349 (23.9)	270 (30.6)	67 (14.9)	12 (9.4)		
RSES	Low	860 (58.9)	473 (53.6)	304 (67.6)	83 (64.8)	41.198	<0.001
	Moderate	412 (28.2)	260 (29.4)	119 (26.4)	33 (25.8)		
	High	189 (12.9)	150 (17.0)	27 (6.0)	12 (9.4)		
GSES	Low	473 (32.3)	193 (21.9)	207 (46.0)	73 (57.1)	130.316	<0.001
	Moderate	638 (43.7)	469 (53.1)	144 (32.0)	25 (19.5)		
	High	350 (24.0)	221 (25.0)	99 (22.0)	30 (23.4)		

Abbreviations: ATMD, Adolescence time management disposition inventory; BMI, body mass index; C1, low distress–low suicidal ideation; C2, moderate distress–moderate suicidal ideation; C3, high distress–high suicidal ideation; CIAS‐R: Revised Chinese internet addiction scale; GSES, general self‐efficacy scale; MEQ‐5, Morningness–eveningness questionnaire; PSQI, Pittsburgh sleep quality index; RSES, Rosenberg self‐esteem scale.

^a^Kruskal–Wallis test.

### 3.3. Multinomial Logistic Regression Analysis of Factors Associated With LP‐PDSI Profiles

The multinomial logistic regression model showed significantly improved fit compared with the intercept‐only model (likelihood ratio *χ* ^2^ = 550.047, df = 50, *p*  < 0.001). Goodness‐of‐fit tests indicated acceptable model performance (Pearson *χ* ^2^ = 2730.991, df = 2868, *p* = 0.966; Deviance *χ* ^2^ = 2022.411, df = 2868, *p*  > 0.999). The Nagelkerke pseudo‐*R* ^2^value was 0.379. No evidence of problematic multicollinearity was observed among the independent variables, with all tolerance values exceeding 0.10 and all VIF values below 5.

Multinomial logistic regression analysis was conducted with LP‐PDSI profiles as the dependent variable (using C1 [low distress–low suicidal ideation] as the reference group) and univariately significant variables as independent variables. The results demonstrated that BMI, institution type, weekly exercise frequency, academic performance, parental relationship, sleep quality (PSQI), chronotype (MEQ‐5), internet addiction level (CIAS‐R), time management skills (ATMD), self‐esteem level (RSES), and self‐efficacy level (GSES) were significantly associated with LP‐PDSI profiles among nursing students.

As shown in Table 5, the multinomial logistic regression identified significant factors associated with profile membership. Contextual and physiological factors, such as higher BMI and enrollment in an undergraduate institution, were uniquely associated with membership in the C3 profile. In contrast, behavioral factors, particularly engaging in weekly exercise (≥1 time) and having a good parental relationship, were associated with lower odds of belonging to C3. Several factors were commonly associated with membership in both the C2 and C3 profiles, including poor sleep quality and higher levels of internet addiction. Conversely, moderate self‐efficacy emerged as a factor associated with lower odds of membership in both C2 and C3 profiles. Factors such as better time management skills and higher self‐esteem were specifically associated with a reduced likelihood of belonging to the C2 profile, but not the C3 profile. The odds ratios (ORs) and confidence intervals (CIs) for significant factors are presented in Table 5, while the complete multinomial logistic regression results for all variables are provided in Table S1.

**Table 5:** Significant factors associated with the profile membership identified by multinomial logistic regression analysis.

Variables_(Ref.)_		C2 vs. C1				C3 vs. C1			
		*p*‐Value	OR	95% CI		*p*‐Value	OR	95% CI	
Intercept		0.366	—	—	—	0.063	—	—	—
BMI		0.300	1.025	0.978	1.073	0.026	1.092	1.010	1.180
Institution type _(Vocational college)_	Undergraduate college	0.379	1.129	0.862	1.479	0.001	2.478	1.467	4.186
Weekly exercise frequency _(0 times)_	≥5 times	0.376	0.795	0.478	1.322	0.050	0.407	0.166	0.999
	3–4 times	0.344	0.806	0.516	1.260	0.017	0.393	0.182	0.846
	1–2 times	0.305	0.828	0.577	1.188	0.033	0.529	0.295	0.949
Academic performance _(Poor)_	Good	0.010	0.443	0.238	0.826	0.972	0.983	0.373	2.588
	Moderate	0.059	0.642	0.406	1.018	0.686	0.861	0.418	1.775
Parental relationship _(Poor)_	Good	0.803	0.935	0.551	1.586	0.042	0.465	0.222	0.974
	Moderate	0.352	1.310	0.742	2.312	0.428	0.727	0.330	1.600
PSQI _(Poor sleeper)_	Good sleeper	<0.001	0.508	0.391	0.660	<0.001	0.196	0.117	0.327
MEQ‐5 _(Evening type)_	Morning type	0.132	1.591	0.870	2.912	0.001	4.662	1.848	11.763
	Neutral type	0.172	1.229	0.914	1.653	0.005	2.176	1.265	3.743
CIAS‐R _(Normal)_	Addiction	<0.001	4.144	2.646	6.489	<0.001	11.655	5.992	22.670
	Overuse	<0.001	2.952	2.128	4.094	<0.001	10.347	6.076	17.622
ATMD _(Low)_	High	0.002	0.535	0.362	0.791	0.064	0.477	0.218	1.044
	Moderate	0.012	0.677	0.500	0.917	0.285	0.762	0.464	1.253
RSES _(Low)_	High	<0.001	0.368	0.219	0.618	0.645	0.817	0.346	1.929
	Moderate	0.033	0.719	0.531	0.973	0.530	0.844	0.497	1.433
GSES _(Low)_	High	0.176	0.764	0.518	1.128	0.069	0.549	0.288	1.048
	Moderate	<0.001	0.397	0.294	0.536	<0.001	0.218	0.124	0.383

*Note:* Only statistically significant factors associated with latent profile membership are presented in this table. The complete multinomial logistic regression results for all variables included in the model are provided in Table S1.

Abbreviations: ATMD, Adolescence time management disposition inventory; BMI, body mass index; C1, low distress–low suicidal ideation; C2, moderate distress–moderate suicidal ideation; C3, high distress–high suicidal ideation; CI, confidence interval; CIAS‐R, Revised Chinese internet addiction scale; GSES, general self‐efficacy scale; MEQ‐5, Morningness–eveningness questionnaire; OR, odds ratio; PSQI, Pittsburgh sleep quality index; RSES, Rosenberg self‐esteem scale.

## 4. Discussion

This study identified three distinct latent profiles of psychological distress and suicidal ideation among nursing students: the low distress–low suicidal ideation (C1, 60.4%), the moderate distress–moderate suicidal ideation (C2, 30.8%), and the high distress–high suicidal ideation (C3, 8.8%). The concurrent escalation of distress and suicidal ideation across profiles aligns with the cognitive model of suicide [59]. Notably, although C3 represented the smallest profile, its elevated scores across all dimensions highlight this profile as warranting particular attention in future screening and preventive efforts.

### 4.1. Latent Profiles of Psychological Distress and Suicidal Ideation

The three‐profile solution identified in this study parallels latent profile structures observed in previous research on mental health heterogeneity among both medical and nonmedical students [39]. Our findings of the profiles demonstrate that heterogeneous patterns of co‐occurring psychological distress and suicidal ideation rather than a single uniform severity pattern. The C1 profile exhibited the lowest levels across all indicators, consistent with studies indicating most university students maintain relatively stable mental health [60]. This profile may be appropriate for universal preventive mental health education and enhanced psychological resilience training to sustain their well‐being. C2 represented a profile with co‐occurring moderate psychological distress and suicidal ideation. In this sample, moderate distress was not invariably accompanied by severe suicidal ideation. Nevertheless, students in this profile should not be overlooked, as longitudinal evidence indicates that individuals with persistent moderate psychological distress face significantly elevated future suicide risk [61]. Prior study has associated nursing students’ clinical and academic demands with psychological distress and suicide‐related outcomes [17], highlighting the importance of early screening and stress‐management strategies. However, these pathways were not examined in the present study. The smallest profile, C3, demonstrated the highest levels across all indicators, comprising 8.8% of the sample. This proportion exceeds the 3.3% prevalence of a “high suicidal ideation” profile reported among general university students [39], suggesting potential vulnerabilities among nursing students characterized by this profile [13, 14]. The co‐occurrence of severe distress and suicidal ideation in C3 is compatible with the stress–diathesis model, which theorizes that suicide‐related risk may emerge through interactions between environmental stressors (e.g., clinical trauma, academic pressure) and individual vulnerabilities (e.g., poor coping skills) [26, 27]. Critically, while students in the C3 profile showed comparable levels of positive suicidal ideation to C2, they exhibited significantly higher NSI, suggesting the need for enhanced screening attention and preventive support among students presenting with this pattern.

### 4.2. Key Factors Associated With Latent Profiles

Students experiencing psychological distress can have positive mental health [62]. Multinomial logistic regression identified key factors significantly associated with profile membership, including BMI, institution type, exercise frequency, academic performance, parental relationships, sleep quality, chronotype, internet addiction, time management, self‐esteem, and self‐efficacy. These findings elucidate the heterogeneity of mental health patterns among nursing students and may inform targeted screening and preventive strategies.

Higher BMI was associated with membership in the high distress–high suicidal ideation profile (C3) (OR = 1.092, 95% CI: 1.010–1.180). This aligns with evidence linking obesity to elevated psychological distress in young adults [63]. Prior study has proposed body image dissatisfaction as one possible explanation for associations between higher BMI and psychological distress [64, 65]. However, this pathway was not evaluated in the present study. Institution type was significantly associated with profiles. Undergraduate nursing students exhibited 2.478‐fold higher odds of C3 membership compared to vocational college peers (95% CI: 1.467–4.186). Prior studies have associated demanding academic and clinical requirements with psychological distress [39, 66]. However, differences in program demands were not measured in the present study. Weekly exercise frequency was associated with a lower likelihood of C3 membership. Students exercising ≥ 1 time/week had significantly lower C3 odds than sedentary peers (all *p* ≤ 0.05, OR < 1). This finding is consistent with previous evidence suggesting that physical activity is associated with lower levels of anxiety, depression, and stress symptoms [67] and is associated with lower suicidal ideation [68]. Prior literature has proposed several neurobiological and coping‐related explanations for the association between physical activity and mental health [69]. However, these mechanisms were not examined in the present study. Good academic performance was associated with lower odds of moderate distress–moderate suicidal ideation (C2) membership relative to the low distress–low suicidal ideation (C1) (OR = 0.443, 95% CI: 0.238–0.826). This corroborates studies linking academic achievement to lower anxiety, depression, stress, and suicide risk [70, 71]. Potential explanations discussed in previous studies include differences in self‐efficacy and achievement‐related anxiety; however, these pathways were not evaluated in the present study. Good parental relationships were associated with 53.5% lower odds of C3 membership (OR = 0.465, 95% CI: 0.222–0.974). This finding is consistent with previous literature linking more favorable family relationships with better mental health among young adults [72, 73]. Emotional resilience and adaptive coping have been discussed as possible correlates of supportive family environments [74], but these mechanisms were not tested in the present study. Roommate relationships were not statistically significant, which should not be interpreted as evidence that roommate or broader peer support is unimportant.

Sleep quality showed comparatively strong adjusted associations with profile membership. Relative to poor sleepers in C1, individuals with good sleep quality (PSQI ≤ 5) exhibited 49.2% lower odds of belonging to C2 (moderate distress–moderate suicidal ideation) and 80.4% lower odds of belonging to C3 (high distress–high suicidal ideation), highlighting the association between sleep quality and profile membership, particularly for students in C3. This aligns with extensive evidence linking poor sleep quality to higher levels of depression, anxiety, stress, and suicide risk [75–77]. Previous studies have proposed reciprocal associations between sleep quality and psychological distress involving emotion regulation, negative cognition, circadian processes, and coping [75, 77]. These pathways were not examined in the present study. Furthermore, previous research has suggested that poor sleep quality is associated with impaired emotional regulation and elevated suicide risk, potentially through pathways involving depression and anxiety [78]. Chronotype also demonstrated significant associations. Notably, both morning chronotype (OR = 4.662, 95% CI: 1.848–11.763) and neutral chronotype (OR = 2.176, 95% CI: 1.265–3.743) were associated with higher odds of C3 membership compared to evening types. This finding contrasts with prior studies in general university students [43] and nursing students [79, 80], which typically associate evening types with poorer mental health. Several factors may contribute to this unexpected finding. For example, morning chronotypes may differ from evening types in sleep schedules and sleep‐related behaviors, which could influence sleep quality and psychological well‐being [81]. However, these potential mechanisms were not directly examined in the present study. Residual confounding, measurement or categorization effects, unmeasured sleep‐related factors, or sample‐specific characteristics may also partly explain this association. Regarding internet use behaviors, both internet overuse and addiction were strongly associated with membership in both the C2 and C3 profiles relative to C1. Crucially, internet addiction was strongly associated with membership in the C3 profile (OR = 11.655, 95% CI: 5.992–22.670), highlighting the importance of early screening and support strategies addressing problematic internet use behaviors. Previous studies have linked problematic internet use, social isolation, exposure to harmful online content, and suicidality [82–86]. However, these mechanisms were not evaluated in the present study. Stronger time management skills (both high and moderate levels) were significantly associated with a lower likelihood of membership in the C2 profile. This finding is consistent with previous research on time management disposition [87, 88]. Previous studies have also linked time management skills with broader psychological characteristics, including subjective well‐being [87], self‐regulation [88], interpersonal functioning [89], and meaning in life [87]. However, whether these factors explain the association between time management skills and psychological distress profiles requires further investigation. Stronger self‐esteem (both high and moderate levels) was significantly associated with a lower likelihood of C2 profile membership. These findings are consistent with prior studies reporting associations between higher self‐esteem and better mental health [90, 91] and more adaptive coping [92, 93]. Previous research has suggested that self‐esteem may be associated with suicide risk, with lower self‐esteem often observed alongside greater depressive symptoms, anxiety, and suicidal ideation [94, 95]. Its protective capacity may be overwhelmed when other adverse psychological conditions (e.g., severe depression) exceed a critical threshold [96]. These findings are also broadly consistent with the cognitive model of suicide, which proposes that interaction between low self‐worth and negative emotions may contribute to suicidal ideation [29]. However, self‐esteem should be interpreted only as a correlate of profile membership in the present study, and the mechanisms underlying this association cannot be determined. Finally, self‐efficacy showed a differential pattern of association with profile membership. Moderate self‐efficacy was significantly associated with lower odds of both C2 (OR = 0.397, 95% CI: 0.294–0.536) and C3 (OR = 0.218, 95% CI: 0.124–0.383) membership. Individuals with low self‐efficacy often perceive less control over negative outcomes and may be more likely to engage in passive coping strategies (e.g., avoidance, self‐blame), which have been associated with depression, anxiety, and suicide risk. In contrast, higher self‐efficacy has been associated with greater use of active coping strategies (e.g., help‐seeking, problem‐solving) [97, 98]. Furthermore, self‐efficacy has been associated with better mental health through its relationship with emotion regulation and stress management [99, 100] and participates in a mutually reinforcing cycle with psychological well‐being (where mental health bolsters self‐efficacy, and vice versa) [101], which may be associated with lower suicide risk [102, 103]. Interestingly, this study did not observe a significant association between high self‐efficacy and C2/C3 membership, contrasting with the lower likelihood of C2/C3 membership observed among students with moderate self‐efficacy. Although high self‐efficacy is generally considered beneficial for psychological adjustment, the absence of a significant association in this study may reflect categorization effects, residual confounding, or sample‐specific characteristics. One possible interpretation is that higher levels of self‐efficacy may not uniformly translate into adaptive coping under substantial environmental demands, as excessive confidence may sometimes lead to discrepancies between perceived capabilities and actual demands [104]. However, this interpretation remains tentative and warrants further investigation.

## 5. Limitations and Future Research

Several limitations should be acknowledged. First, the cross‐sectional design prevents causal inferences. Second, the use of convenience sampling from two medical institutions in Anhui Province and the exclusive inclusion of nursing students may limit the generalizability of our findings. Although nursing students represent an important population because of their unique academic and clinical stressors, their sociodemographic characteristics, educational experiences, and cultural contexts may differ from students in other disciplines, educational settings, or geographic regions. Moreover, variations in mental health awareness, stigma, help‐seeking behaviors, and social norms may influence the expression and reporting of psychological symptoms across different contexts. Therefore, the latent profiles identified in this study should be interpreted as context‐specific statistical patterns rather than universally applicable categories. Third, participants were recruited from intact classes within two institutions; however, class‐level identifiers were not retained in the final analytical dataset, preventing direct assessment or adjustment for potential class‐level clustering. Although institution type and grade information were available, they could not adequately capture shared classroom environments. Students within the same educational setting may share academic demands, clinical training experiences, and institutional resources, potentially introducing correlations among observations. Fourth, variables entered into the multinomial logistic regression were selected primarily on the basis of statistical significance in the univariate analyses. Although this strategy was used to limit model complexity, it may have excluded theoretically relevant covariates that were not statistically significant in univariate comparisons. Consequently, residual confounding cannot be excluded, and the multivariable findings should be interpreted as exploratory, mutually adjusted associations rather than as evidence of independent risk or protective effects. Fifth, several potentially relevant factors, such as clinically diagnosed mental health conditions and family history of mental illness, were not measured, which may have resulted in residual confounding. Sixth, although multiple statistical criteria were considered to determine the optimal profile solution, latent profiles are sample‐dependent statistical classifications and may vary across samples. Seventh, reliance on self‐reported measures may introduce reporting bias, although anonymity procedures were implemented to reduce this concern.

Future research should prioritize: (1) longitudinal, multicenter, and cross‐cultural studies involving diverse student populations across different educational settings and regions to validate the identified profiles and examine their stability and generalizability; (2) studies incorporating comprehensive clinical, biological, and contextual information (e.g., mental health diagnoses, family history, biomarkers, and sociocultural factors) to reduce residual confounding and improve risk assessment; (3) studies collecting detailed hierarchical information (e.g., class‐level identifiers) and applying multilevel or cluster‐adjusted analytical approaches to account for potential clustering effects; and (4) studies evaluating profile‐informed preventive strategies through randomized controlled trials, while incorporating objective and multimethod assessments where feasible to overcome limitations associated with self‐reported measures.

## 6. Conclusion

This study identified three distinct latent profiles of psychological distress and suicidal ideation among nursing students: low distress–low suicidal ideation (60.4%), moderate distress–moderate suicidal ideation (30.8%), and high distress–high suicidal ideation (8.8%). The high distress–high suicidal ideation profile (C3) exhibited the highest levels across psychological distress and suicidal ideation indicators and was associated with specific contextual and behavioral factors, including higher BMI, undergraduate institution, morning/neutral chronotypes, and poor parental relationship, whereas students who exercising ≥ 1 time per week showed a lower likelihood of membership in this profile. Factors associated with membership in the moderate distress–moderate suicidal ideation and high distress–high Ssicidal ideation profiles (C2/C3) included poor sleep quality, internet overuse/addiction, and moderate self‐efficacy, whereas good academic performance, better time management skills (moderate/high), and self‐esteem were specifically associated with a lower likelihood of membership in the moderate distress–moderate suicidal ideation profile (C2). These findings highlight the heterogeneity of psychological distress and suicidal ideation patterns among nursing students and suggest the potential value of profile‐informed screening approaches that consider potentially relevant factors (e.g., sleep quality, internet use behaviors, exercise frequency, time management, self‐esteem, and self‐efficacy) and contextual factors (e.g., institution type, BMI, and parental relationships).

## 7. Relevance for Clinical Practice

This study uncovers distinct mental health profiles and specific associated factors among nursing students—who will soon become clinical caregivers. These findings may inform nurse educators, clinical preceptors, and mental health professionals when considering screening and preventive support in educational settings. Specifically, the identified profiles may facilitate the recognition of students who may benefit from additional psychological support, including support strategies that consider potentially modifiable factors such as sleep quality, internet use behaviors, time management, self‐esteem, and self‐efficacy. Supporting students’ psychological resilience and mental well‐being may contribute to healthier learning environments and future healthcare workforce well‐being.

## Author Contributions

**Anshi Wang**: conceptualization, data collection, analysis, funding acquisition, writing – original draft, writing – review and editing. **Jingjing Wan**: data collection, analysis, writing – original draft. **Qin Yang**: data collection, analysis, funding acquisition, writing – original draft. **Lijun Zhu, Liying Wen, Weiwei Chang**, **Yu Zhu**: data collection, writing – original draft. **Yan Chen**: conceptualization, project administration, funding acquisition, writing – review and editing. **Yuelong Jin**: conceptualization, supervision, writing – review and editing. **Yingshui Yao**: conceptualization, supervision, funding acquisition, writing – review and editing.

## Funding

This work was supported by the Projects of the Department of Education of Anhui Province (Grants KJ2020ZD69, 2022jyxm1699, 2023cxtd170, and DTR2024031), the Projects of the Anhui Federation of Social Sciences (Grant 2021CX105), and the Projects of Wannan Medical College (Grant WK2024ZQNZ16).

## Ethics Statement

This study strictly followed the Declaration of Helsinki guidelines and was approved by the Ethics Committee of School of Public Health, Wannan Medical College (Approval No. LL‐WK2024ZQNZ16).

## Consent

All participants provided informed consent prior to participation.

## Conflicts of Interest

The authors declare no conflicts of interest.

## Supporting Information

Additional supporting information can be found online in the Supporting Information section.

## Supporting information


**Supporting Information** Table S1 presents the complete results of the multinomial logistic regression analysis of factors associated with latent profile membership among nursing students.

## Data Availability

The datasets used and analyzed during the current study are available from the corresponding author upon reasonable request.
