# Supplementary material for: Latent Profiles of Psychological Distress and Suicidal Ideation Among Nursing Students: A Cross‐Sectional Study of Associated Factors
Source: Depress Anxiety. 2026 Oct 19;2026:9138758. doi: 10.1155/da/9138758 (PMC13679096; doi:10.1155/da/9138758)
Supplement: Supplementary file 1 [file DA-2026-9138758-s001.docx]

| **Supplementary Table S1 Full multinomial logistic regression analysis of factors associated with the profile membership among nursing students** | | | | | | | | | | | | | | |
| --- | --- | --- | --- | --- | --- | --- | --- | --- | --- | --- | --- | --- | --- | --- |
| **Variables _(Ref.)_** | | **C2 vs C1** | | | | | |  | **C3 vs C1** | | | | | |
|  |  | **B** | **SE** | **P** | **OR** | **95% CI** | |  | **B** | **SE** | **P** | **OR** | **95% CI** | |
| **Intercept** |  | 0.657 | 0.727 | 0.366 | - | - | - |  | -2.177 | 1.170 | 0.063 | - | - | - |
| **BMI** |  | 0.024 | 0.024 | 0.300 | 1.025 | 0.978 | 1.073 |  | 0.088 | 0.040 | 0.026 | 1.092 | 1.010 | 1.180 |
| **Gender _(Female)_** | Male | 0.080 | 0.193 | 0.679 | 1.083 | 0.742 | 1.580 |  | 0.536 | 0.301 | 0.074 | 1.710 | 0.948 | 3.082 |
| **Only-child status _(No)_** | Yes | 0.145 | 0.165 | 0.380 | 1.156 | 0.836 | 1.597 |  | 0.287 | 0.268 | 0.285 | 1.332 | 0.788 | 2.253 |
| **Smoking status _(Yes)_** | No | -0.397 | 0.362 | 0.273 | 0.672 | 0.331 | 1.367 |  | -0.580 | 0.521 | 0.265 | 0.560 | 0.202 | 1.554 |
| **Institution type _(Vocational college)_** | Undergraduate college | 0.121 | 0.138 | 0.379 | 1.129 | 0.862 | 1.479 |  | 0.907 | 0.267 | 0.001 | 2.478 | 1.467 | 4.186 |
| **Weekly exercise frequency _(0 times)_** | ≥5 times | -0.230 | 0.260 | 0.376 | 0.795 | 0.478 | 1.322 |  | -0.899 | 0.458 | 0.050 | 0.407 | 0.166 | 0.999 |
|  | 3-4 times | -0.216 | 0.228 | 0.344 | 0.806 | 0.516 | 1.260 |  | -0.935 | 0.391 | 0.017 | 0.393 | 0.182 | 0.846 |
|  | 1-2 times | -0.189 | 0.184 | 0.305 | 0.828 | 0.577 | 1.188 |  | -0.637 | 0.298 | 0.033 | 0.529 | 0.295 | 0.949 |
| **Academic performance _(Poor)_** | Good | -0.814 | 0.318 | 0.010 | 0.443 | 0.238 | 0.826 |  | -0.107 | 0.494 | 0.972 | 0.983 | 0.373 | 2.588 |
|  | Moderate | -0.442 | 0.235 | 0.059 | 0.642 | 0.406 | 1.018 |  | -0.149 | 0.369 | 0.686 | 0.861 | 0.418 | 1.775 |
| **Relationship with roommates _(Poor)_** | Good | -0.202 | 0.271 | 0.456 | 0.817 | 0.480 | 1.390 |  | -0.801 | 0.422 | 0.058 | 0.449 | 0.196 | 1.028 |
|  | Moderate | 0.206 | 0.307 | 0.502 | 1.229 | 0.673 | 2.242 |  | 0.111 | 0.457 | 0.809 | 1.117 | 0.456 | 2.737 |
| **Parental relationship _(Poor)_** | Good | -0.067 | 0.270 | 0.803 | 0.935 | 0.551 | 1.586 |  | -0.766 | 0.377 | 0.042 | 0.465 | 0.222 | 0.974 |
|  | Moderate | 0.270 | 0.290 | 0.352 | 1.310 | 0.742 | 2.312 |  | -0.319 | 0.403 | 0.428 | 0.727 | 0.330 | 1.600 |
| **PSQI _(Poor sleeper)_** | Good sleeper | -0.677 | 0.134 | < 0.001 | 0.508 | 0.391 | 0.660 |  | -1.632 | 0.262 | < 0.001 | 0.196 | 0.117 | 0.327 |
| **MEQ-5 _(Evening type)_** | Morning type | 0.464 | 0.308 | 0.132 | 1.591 | 0.870 | 2.912 |  | 1.539 | 0.472 | 0.001 | 4.662 | 1.848 | 11.763 |
|  | Neutral type | 0.207 | 0.151 | 0.172 | 1.229 | 0.914 | 1.653 |  | 0.778 | 0.277 | 0.005 | 2.176 | 1.265 | 3.743 |
| **CIAS-R _(Normal)_** | Addiction | 1.422 | 0.229 | < 0.001 | 4.144 | 2.646 | 6.489 |  | 2.456 | 0.339 | < 0.001 | 11.655 | 5.992 | 22.670 |
|  | Overuse | 1.082 | 0.167 | < 0.001 | 2.952 | 2.128 | 4.094 |  | 2.337 | 0.272 | < 0.001 | 10.347 | 6.076 | 17.622 |
| **ATMD _(Low)_** | High | -0.626 | 0.200 | 0.002 | 0.535 | 0.362 | 0.791 |  | -0.741 | 0.400 | 0.064 | 0.477 | 0.218 | 1.044 |
|  | Moderate | -0.390 | 0.155 | 0.012 | 0.677 | 0.500 | 0.917 |  | -0.271 | 0.254 | 0.285 | 0.762 | 0.464 | 1.253 |
| **RSES _(Low)_** | High | -1.000 | 0.265 | < 0.001 | 0.368 | 0.219 | 0.618 |  | -0.202 | 0.438 | 0.645 | 0.817 | 0.346 | 1.929 |
|  | Moderate | -0.330 | 0.154 | 0.033 | 0.719 | 0.531 | 0.973 |  | -0.169 | 0.270 | 0.530 | 0.844 | 0.497 | 1.433 |
| **GSES _(Low)_** | High | -0.269 | 0.199 | 0.176 | 0.764 | 0.518 | 1.128 |  | -0.599 | 0.330 | 0.069 | 0.549 | 0.288 | 1.048 |
|  | Moderate | -0.923 | 0.153 | < 0.001 | 0.397 | 0.294 | 0.536 |  | -1.523 | 0.286 | < 0.001 | 0.218 | 0.124 | 0.383 |
| C1: Low Distress-Low Suicidal Ideation; C2: Moderate Distress-Moderate Suicidal Ideation; C3: High Distress-High Suicidal Ideation; BMI: body mass index; PSQI: Pittsburgh Sleep Quality Index; MEQ-5: Morningness-Eveningness Questionnaire; CIAS-R: Revised Chinese Internet Addiction Scale; ATMD: Adolescence Time Management Disposition Inventory; RSES: Rosenberg Self-Esteem Scale; GSES: General Self-Efficacy Scale; OR: odds ratio; CI: confidence interval | | | | | | | | | | | | | | |
